# New expectations: Pediatric cochlear implantation in Japan

**DOI:** 10.1179/1467010013Z.00000000079

**Published:** 2013-03

**Authors:** Janette Oliver

**Affiliations:** VP Clinical Applications Japan Cochlear Limited, Tokyo, Japan

**Keywords:** Japan, Health care, Neonatal hearing screen, Pediatric cochlear implantation, Funding, Access

## Abstract

**Funding for cochlear implants:**

The Japanese health-care system provides universal health coverage for the entire 127 million population of Japan. This includes all aspects of cochlear implantation, from diagnosis to implantation to mapping and habilitation aftercare. Japan has the third largest developed economy; however, the uptake rate for cochlear implants is lower than that of countries with similar economic status. Japan has an uptake rate of approximately 1% of potentially suitable subjects of all ages, compared with 5.6% in the USA.

**Cochlear implant provision for children:**

In Japan, about 55% of cochlear implant recipients are children of less than 18 years of age. This represents an increase of 20% in the last 10 years, with a relative increase in the numbers of children receiving implants compared with the numbers of adults. However, only 3–4% of children under the age of 3 years are being implanted at less than 18 months of age. This is in accordance with the Japanese ENT Academy's guidelines, which currently puts the minimum age limit for implants in children at 18 months.

**Neonatal screening:**

For hearing loss was first piloted nationally in Japan in 2000. Funding for screening subsequently stopped in 2005, though the national treasury provided a further 2 years' funding. Since 2007 local government organizations have been given responsibility to support these screening programs, but there remains considerable variation in funding between different prefectures. In one prefecture, Okayama, 95% of babies were screened and followed up for 2 years. However, the support system for children who need further diagnostic testing after screening remains insufficient.

**Referral:**

When diagnosed, children with hearing loss are referred for counselling, hearing aids and habilitation. The responsibility for these is divided between the Ministry of Health and Welfare (including surgery, device programming, and therapy) and the Ministry of Education. Schools for the deaf and preschool hearing impaired education centers have had most of the responsibility for early intervention, educational choices and referral for cochlear implantation. In the past 98% of schools for children with hearing loss have used communication methods relying mostly on visual cues. In recent years, however, there has been a shift toward ‘inclusive’ mainstream education. Between 2008 and 2011 the number of children with cochlear implants in special needs schools increased to 16%. It is now estimated that 67% of children with cochlear implants may now be in mainstream schools.

There is still the need for support services for these implanted children attending mainstream schools, with adequate provision of resources.

**Conclusion:**

Cochlear Implantation has had a significant role in changing the medical management and education of children in Japan with hearing loss. Much remains to be done, though the situation has greatly improved in recent years and continues to do so.

## Introduction

Internationally, the opportunities for the development of hearing, listening, and spoken language are greater than ever before. Universal newborn hearing screening has become instrumental in the diagnosis of hearing loss much earlier than in the past. Consequently, babies and very young children can be fitted, even in infancy, with state of art hearing technology *including cochlear implants*.

The positive benefits of early diagnosis and cochlear implantation are well documented in the literature (Dettman *et al*., 2007; Philips *et al*., 2009; Ching *et al*., 2009; Niparko *et al*., 2010; Yoon, 2011; Geers *et al*., 2011; Kral and Sharma, 2012). Parents and families have new expectations.

Due to these positive benefits and new expectations, changes are required in policy and service delivery across the board, but especially in the areas of habilitation options and educational access to accommodate the ‘new *deaf* child’.

Cochlear implantation is now being considered by increasing numbers of families in Japan who are seeking optimal listening and spoken language outcomes for their children. Japan's policy-makers, educators and hearing health service providers, like their overseas counterparts, are now faced with a shifting paradigm that underscores the need for innovation.

## The medical/regulatory landscape in Japan

Japan's health-care system is characterized by universal coverage for the population of approximately 127 million people. Japan boasts the longest life expectancy at birth and has the lowest infant mortality rate of any country in the OECD (Organization for Economic Co-operation & Development). Although there is strong government regulation of health-care financing and the operation of health insurance, control of the delivery of care is left largely to the medical profession.

All aspects of the cochlear implant journey from diagnosis and referral to implantation, mapping and habilitation aftercare theoretically fall under the classification of medical care in Japan.

(Re) habilitation for cochlear implantation has been historically included in the same classification as those rehabilitative interventions for cerebral vascular diseases (stroke), such as aphasia, cognitive communication deficits, apraxia, and dysarthria.

There is no distinct profession of audiology in Japan. General Practice ENT doctors perform many of the aspects of hearing health care associated with a professional audiology practice in North America and the UK, such as otoscopy and tympanometry, while audiometry and the fitting of hearing technology is most often conducted by clinicians.

The cochlear implant clinician's role in Japan is filled by Speech/hearing/language therapists who are tasked with a very broad scope of practice. Clinicians in Japan may be required to deal with everything from cochlear implant programming and hearing aid fitting to aphasia, stuttering and feeding problems, and sometimes all in the same working day. Relatively few clinical professionals have the opportunity to specialize solely in hearing healthcare and cochlear implantation.

Traditionally, speech therapists in Japan have been classified as paramedical staff under the direct instruction of physicians. It took nearly 30 years to reach consensus in defining the status of speech therapists before the 1997 National Law for Speech Therapists was passed (Iitaka, 2006). As a result of these negotiations Japanese speech therapists were able to work without directives from physicians with the *exception* of training for dysphagic patients and rehabilitative evaluations and training for patients having *cochlear implantation.*

In practice of course, these directives are largely nominal in 2012; but nevertheless the requirement for ‘medical supervision’ places some practical constraints on potential models for cochlear implant services and long-term aftercare solutions for the steadily increasing number of cochlear implant recipients across the country.

Many cochlear implant programs around the world are currently facing the challenge of long-term management of a rapidly growing client base through networking and referrals to non-medical satellite centers or education programs, and implementation of telepractice and self programming services. As cochlear implantation becomes a mainstream intervention, programs in Japan will face similar challenges and this may require some reinterpretation of existing practice regulations.

While the number of children and adults receiving cochlear implants in Japan continues to rise, it is salutary to consider that the 1% uptake rate of cochlear implants by potentially suitable candidates of all ages remains considerably lower than in other developed countries, for example, 5.6% in the USA*.*

The current trend is toward younger age of implantation in Japan. However, if we look at the critical language learning period of 0–3 years of age it is clear that Japan is more conservative in acting on the ‘younger is better’ message evident from the research. Only 3–4% of the under three's are being implanted at 18 months of age or younger (based on the manufacturer's estimates). But this is completely in accordance with the current guidelines from the Japanese ENT Academy which state 18 months as the lower age limit for candidacy.

Age at implantation is also impacted by identification and early intervention issues. Wada *et al*. (2004) suggest that one of the most pressing issues in the timely management of infant hearing loss in Japan is the need for improvement in both the early intervention systems and diagnostic follow-up to identification. While progress has been made in the intervening period, this evaluation could still be said to hold certain validity today.

## Identification of hearing loss

The initiation of screening of newborns for hearing loss in Japan came about largely as a result of the establishment of a nationwide pilot study from 2000 with grants from the Ministry of Labor, Health and Welfare (MLHW). Funding from the MLHW was terminated in March 2005, but was supported for a further 2 years by the national treasury. Since March 2007, however, local governments have had to shoulder the responsibility for supporting screening programs. So although screening has spread nationwide, there is considerable variability across prefectures due to the dependence on local funding.

Kobayashi and Inadera (2011) report that a 2005 survey questionnaire by the Japan Association of Obstetricians and Gynecologists, found that 62% overall of newborn babies were given audiology and medical assessment compared to an overall 95% in the USA in 2007.

Reports from some individual prefectures, however, show results comparable to the USA. Fukushima *et al*. (2008) report that 95% of infants born in Okayama Prefecture between June 2001 and March 2005 were screened and followed up for at least 2 years.

The implementation of neonatal hearing screening and subsequent follow-up in Japan is further complicated by the traditional practice of ‘back to home’ childbirth (Fukushima *et al*., 2008). *Satogaeri bunben* is a Japanese rite where the pregnant mother returns to her family home for the delivery and stays with her parents and family members for support and to rest after the birth for a few weeks.

This cultural tradition potentially means that many mothers and infants are moving from one part of Japan to another in the perinatal period. This may often prove an obstacle to follow-up for children identified as ‘at risk’ by the neonatal screening program, as each of the 47 prefectures have independent systems in place.

## Referral for intervention

Following diagnosis, families are referred on for counseling, hearing aid fitting, and habilitation. It is at this point that the path on the cochlear implant journey becomes split between medical and educational fields and their associated bureaucracies of the Ministry of Health & Welfare and the Ministry of Education. ‘Habilitation’ in the form of device programming and therapy remains firmly in the medical camp. Historically there has been relatively little interaction between the cochlear implant program and educational program.

One of the more obvious constraints in Japan is the relatively limited nature of the infrastructure and resources available for early intervention services for children who are deaf or hard of hearing.

As Kobayashi and Inadera (2011) point out in their recent analysis, there is no sufficient support system for children whose families are advised to undertake further diagnostic testing following initial screening. ‘It is necessary for government agencies, medical and educational institutions to communicate together for clarifying their responsibilities in order to support the children with hearing impairment.’

So in the absence of a specialized and widespread early intervention infrastructure, these functions often fall to the schools for the deaf and preschool hearing-impaired education centers in Japan.

These schools and education centers play a key ‘gatekeeper’ role in this critical process and influence referral for cochlear implantation, educational choices, and access to spoken language and mainstream options.

## Education environment

Orientation toward more inclusive educational policies witnessed from international trends has become increasingly evident in Japan over the past decade. The Ministry of Education, Science, Sports and Culture (MEXT) or ‘Monbusho’ is responsible for all education in Japan. Historically, special schools were established completely separately by types of disabilities, such as ‘Schools for the Blind’, ‘Schools for the Deaf’ and ‘Schools for the Intellectually Disabled’. A working group set up by the Central Council for Education in the Ministry of Education, Culture, Sports, Science and Technology to discuss ‘special supportive education’, issued a ‘Midterm Report’ back in 2004, which recommended dual placement and open room concepts.

Dual placement means that children in special schools and special classes are also enrolled in regular classes in regular schools in their community. Open room aims for all children who have special needs to be given support at any time. This is comparable to the system of visiting or itinerant teachers of the deaf found in many countries such as Australia, USA, UK, and Canada. These concepts received relatively limited implementation at that time.

The School Education Law was partially amended and enacted in 2007. Under the new system, there are officially only ‘Schools for Special Needs Education’, and one particular school can accept several disabilities, though in reality many continue to function as category-specific special schools.

There are also resource rooms and special classes in elementary and lower secondary schools.

These schools comprise four levels of departments, namely, kindergarten, elementary, lower secondary and upper secondary departments. (The elementary and the lower secondary are compulsory education.) The cost per student in Schools for Special Needs Education and Schools for the Deaf is about 10 times as high as that in regular schools according to MEXT's official website

Teachers may receive their pre-service training at any university or junior college with a teacher-training course approved by the Monbusho. Most recently qualified teachers have bachelor's degrees rather than technical certificates. There is a system of in-service training which ensures appropriate teacher certification but no formal requirement to have postgraduate qualifications in special education or hearing impairment. Many teachers are assigned to Schools for the Deaf and typically rotated through the system on a 3- to 5-year basis.

**Figure 1:**
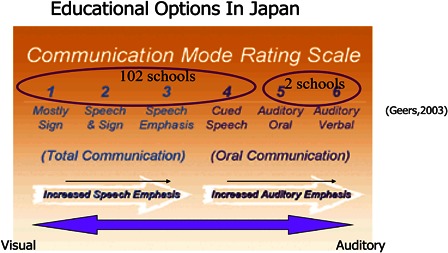
Educational options in Japan (Tono, T. Oral Presentation 12th International Conference on Cochlear Implants in Children, Seattle, WA, 18–20 June 2009 (personal communication).

Communication options available in these special schools has been characterised broadly as follows by Tono T (2009, personal communication) (Fig. 1).

This broad characterization of communication options suggests that 98% (102/104) of schools catering for children with hearing loss employ communication modalities with strong visual emphasis. This raises questions as to whether the increasing numbers of children with high levels of auditory access as a result of cochlear implantation have sufficient options open to them with auditory and spoken language emphasis.

This scenario clearly poses a challenge to families seeking access to auditory-based spoken language options within the special needs education system.

However, it is clear that there has been increasing momentum toward inclusive educational policies over recent years.

MEXT is now pursuing a goal of ensuring the development of an ‘inclusive education system’. In 2010, the Central Council for Education Special Committee of the Future Direction of Special Needs Education agreed in principle that the future direction of special needs education would be toward the establishment of an inclusive education system. Last year saw an amendment to the Basic Law for Persons with Disabilities which was promulgated and enacted in August 2011. The provision related to education states that the Government and the local governments shall take necessary measures to improve and enrich contents and method of education to enable persons with disabilities to receive adequate education in accordance with their age, capacity, and characteristics of disability by educating students with disabilities together with mainstream students to the greatest extent possible.

**Figure 2:**
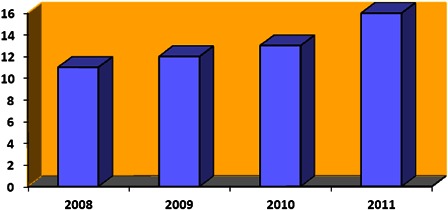
Percentage of the special education population with cochlear implants (data: MEXT).

Data from MEXT tell an interesting story about the changes taking place in the distribution of children with cochlear implants within the school population. During the period from 2008 to 2011 we see an increase in the number of children with cochlear implants to 16% of enrollments in the special needs education system (Fig. 2).

Extrapolating from MEXT enrollment figures for children aged 6–11 years in schools for the deaf, in combination with manufacturers' estimates of pediatric recipients, it is estimated that as many as 67% of children with cochlear implants aged 6–11 years may now be in mainstream schools.

Mainstream placement, however, does not obviate the need for support services, which are dependent upon individual, child-specific considerations (Kral and Sharma, 2012; Cosetti and Waltzman, 2012). Japan will need to look at the equitable provision of resources to support the needs of children with cochlear implants in the mainstream and consider ways to update the special needs educational setting to cater for the ‘new *deaf* child’.

## Parents and families

Many families pursue cochlear implantation in part because they want their children to attend school with their normal hearing peers. (Archbold *et al*., 2006).

All parents want their children to succeed in school, at work, and in whatever they pursue in life. Parents of children who are deaf or hard of hearing are no different. The mainstream school environment is often viewed by many families as a place for their child to continue the journey toward an integrated life in the larger world following cochlear implantation.

Parent groups can be a powerful force for developing, implementing and assessing programs and policies to positively affect the hearing health-care and education of children with hearing loss. In Japan we are witnessing the growth of parent-based groups, which are actively working toward addressing these issues (Oliver and Sorkin, 2010).

These groups are pressing for full disclosure of information and options. Indeed parent participation was specified in the School Education Law amended in 2007. The law provided that the local board of education must hear opinion not only from the educational professionals but also from parents. In 2012 increasing numbers of parents are seeking to make their opinions heard.

Parents want to fully understand the range of educational options in order to be able to make informed choices. Many services for children with hearing loss and their families are now offered internationally because parents learned about their importance and then lobbied governments and service providers to offer those services to their children.

## International trends

In many developed countries cochlear implantation has played a significant role in influencing the medical management of hearing loss and educational choices being made by families, with trends toward the use of spoken language, to mainstream education, and an improvement in early identification and intervention.

In Japan, we are seeing very positive moves toward change and the opportunities for children with cochlear implants are greater than ever before, but as is the case globally there still remains so much more to be done to optimize the benefits of cochlear implantation. With the dedication of hearing healthcare and educational professionals, the hopes and expectations of parents and families and the support of policy-makers, the future is full of promise for children with cochlear implants in Japan.
